# Supplementary material for: Fibroblast Growth Factor 23 Signaling Does Not Increase Inflammation from Pseudomonas aeruginosa Infection in the Cystic Fibrosis Bronchial Epithelium
Source: Medicina (Kaunas). 2023 Sep 9;59(9):1635. doi: 10.3390/medicina59091635 (PMC10538042; doi:10.3390/medicina59091635)
Supplement: Supplementary file 1 [file medicina-59-01635-s001.zip › medicina-2577941-supplementary.pdf]

Supplemental Figure S1

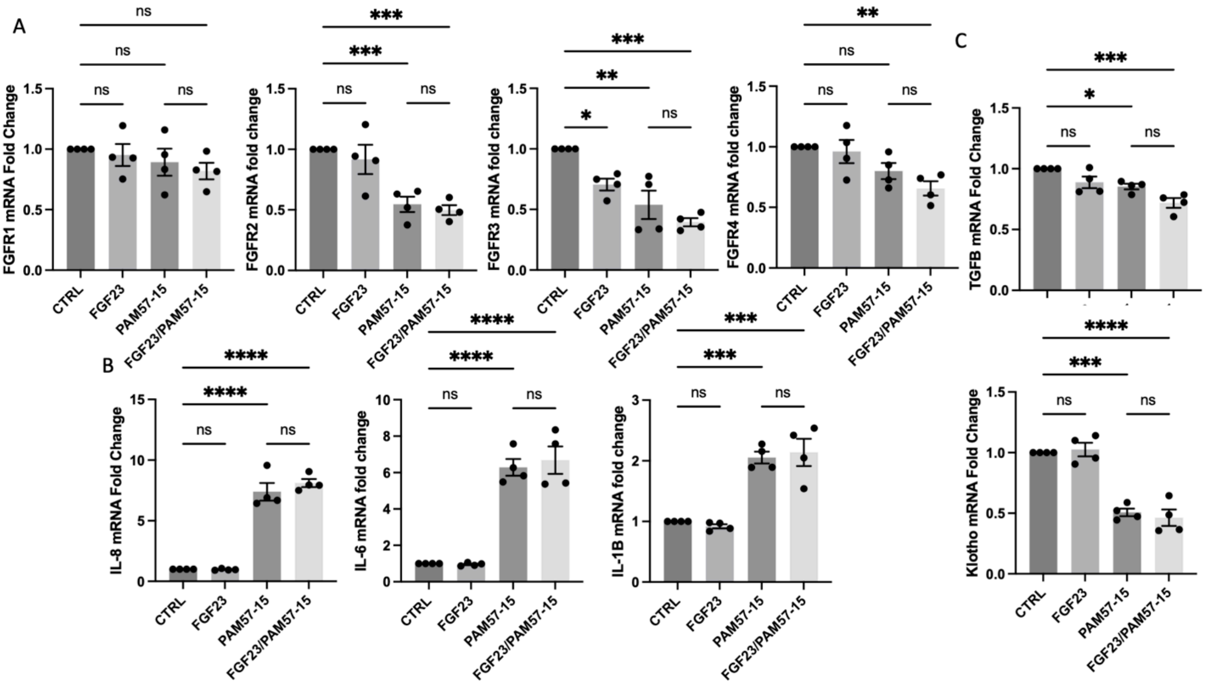

Supplemental Figure S1. FGF23 does not exacerbate pro-inflammatory markers at the transcript level in the PA infected 16HBEs. (**a**) Fold change mRNA levels of FGFRs 1-4 ± FGF23 and PA (PAM57-15). (**b**) Fold change mRNA levels of CXCL8 (IL-8), IL-6, and IL-1β after treatment ± FGF23 ± PA. (**c**) Fold change mRNA levels of TGFβ and klotho after treatment ± FGF23 ± PA infection. Data was represented as fold change mRNA expression with n=4 independent experiments. Statistical analysis was done using a 2-way ANOVA, followed by Tukey's multiple comparisons *post hoc* test. Data are expressed as means ± standard error of the mean (SEM). Differences were considered statistically significant if *P<0.05, ** P<0.01, ***P<0.005, and ****P<0.0001.

Supplemental Figure S2

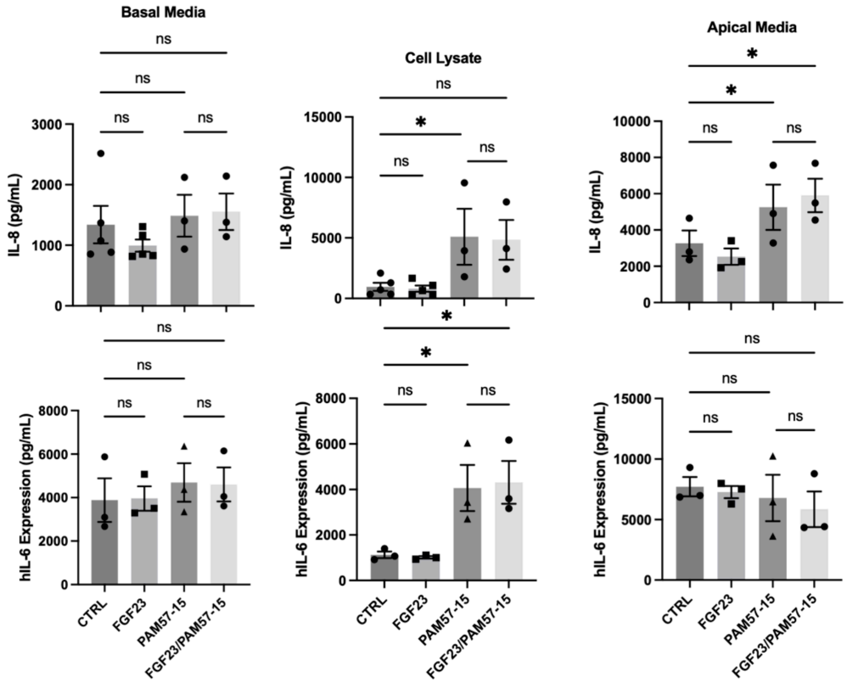

Supplemental Figure S2. FGF23 does not alter pro-inflammatory marker production and secretion in PA-infected 16HBEs. (**a**) Graph showing levels of IL-8 protein expression in the basolateral media, cell lysate, and apical media when 16HBEs are treated ± FGF23 and ± PA infection. (**b**) Graphs showing levels of IL-6 protein expression in the basolateral media, cell lysate, and apical media when 16HBEs are treated ± FGF23 and ± PA infection. Data was represented as expression (pg/mL) with n=3 independent experiments. Statistical analysis was done using a 2-way ANOVA, followed by Tukey's multiple comparisons *post hoc* test. Data are expressed as means ± standard error of the mean (SEM). Differences were considered statistically significant if *P<0.05.
